# Comparison of Goal Achievement during an Early, Intensive Nutrition Intervention Delivered to People with Upper Gastrointestinal Cancer by Telephone Compared with Mobile Application

**DOI:** 10.1155/2024/7841826

**Published:** 2024-03-26

**Authors:** Kate Furness, Catherine E. Huggins, Lauren Hanna, Daniel Croagh, Mitchell Sarkies, Terry P. Haines

**Affiliations:** ^1^Nutrition and Dietetics, Monash Health, Monash Medical Centre, Clayton, Victoria 3168, Australia; ^2^Dietetics, Department of Nursing and Allied Health, Swinburne University of Technology, Hawthorn, Victoria 3122, Australia; ^3^Department of Physiotherapy, School of Primary and Allied Health Care, Faculty of Medicine, Nursing and Health Sciences, Monash University, Frankston, Victoria 3199, Australia; ^4^School of Primary and Allied Health Care, Faculty of Medicine, Nursing and Health Sciences, Monash University, Frankston, Victoria 3199, Australia; ^5^Department of Nutrition, Dietetics and Food, School of Clinical Sciences, Faculty of Medicine Nursing and Health Sciences, Monash University, Clayton, Victoria 3168, Australia; ^6^Upper Gastrointestinal and Hepatobiliary Surgery, Monash Medical Centre, Clayton, Victoria 3168, Australia; ^7^Department of Surgery, School of Clinical Sciences, Faculty of Medicine, Nursing and Health Sciences, Monash University, Clayton, Victoria 3168, Australia; ^8^Centre for Healthcare Resilience and Implementation Science, Australian Institute of Health Innovation, Faculty of Medicine, Health and Human Sciences, Macquarie University, Sydney, NSW 2109, Australia

## Abstract

**Objective:**

This study is aimed at exploring whether the mode of nutrition intervention delivery affected participant goal achievement in a three-arm randomised controlled trial of early and intensive nutrition intervention delivered to upper gastrointestinal cancer patients.

**Methods:**

Newly diagnosed upper gastrointestinal cancer patients were recruited from four tertiary hospitals in Melbourne, Australia. Participants in the intervention groups received a regular nutrition intervention for 18 weeks from an experienced dietitian via telephone or mobile application (app) using behaviour change techniques to assist in goal achievement. Univariate and multiple regression models using STATA determined goal achievement, dose, and frequency of contact between groups. A *p* value <0.05 was considered statistically significant.

**Results:**

The telephone group (*n* = 38) had 1.99 times greater frequency of contact with the research dietitian (95% CI: 1.67 to 2.36, *p* < 0.001) and 2.37 times higher frequency of goal achievement (95% CI: 1.1 to 5.11, *p* = 0.03) compared with the mobile app group (*n* = 36). The higher dose (RR 0.03) of intervention and more behaviour change techniques employed in the telephone group compared with the mobile app group increased participant goal achievement (95% CI: 0.01 to 0.04, *p* < 0.001). *Discussion*. Telephone nutrition intervention delivery led to a higher frequency of goal achievement compared to the mobile app intervention. There was also a higher number of behaviour change techniques employed which may have facilitated the greater goal achievement. Mobile app-based delivery may have poorer acceptance in this population with high levels of withdrawal. *Practice Implications*. We need to ensure that specifically designed technologies for our target populations are fit for purpose, efficacious, and acceptable to both patients and healthcare providers. This trial is registered with ACTRN12617000152325.

## 1. Background

Malnutrition commonly results from cancers of the upper gastrointestinal tract (gastric, oesophageal, and pancreatic) [1–3]. It causes increased chemotherapy toxicities, dose reductions, and incomplete therapy conferring lower quality of life (QoL), higher morbidity, and lower overall survival [1, 4, 5]. Poor access to dietitians prior to the commencement of treatment means that many of these patients receive oncological treatments without nutrition support, exacerbating this malnutrition [6]. To bridge this gap, a three-arm randomised controlled trial was performed to deliver early and intensive nutrition counselling via telephone or mobile application to people with UGI cancers to assess the effect on quality-adjusted life years lived compared to a control usual care group. The detailed protocol and results for the RCT have been described previously [7, 8].

The COVID-19 health pandemic has seen a rapid pivot to the use of eHealth technologies in the provision of healthcare. Electronic health (eHealth) is defined as the delivery of healthcare using information and communication technologies [9]. Mobile health (mHealth) uses mobile technologies such as smart phone applications that enable patients to self-monitor symptoms through biofeedback mechanisms, providing direct interaction with healthcare providers from a distance, and providing reminders, prompts, and alarms to action [10]. eHealth can be delivered via synchronous, asynchronous, or combined methods. Synchronous interventions are delivered in real time, whereas asynchronous interventions are delivered using store and forward methods, meaning that there is no real-time interaction between healthcare provider and patient [11–13].

Complex health-related behaviour change interventions are thought to impact target populations through multiple interacting components [14, 15]. To effectively deliver nutrition care that leads to behaviour change in individuals, trained dietitians use personalised nutrition care plans which centre around goal setting, specifically the setting of goals that are specific, measurable, action-based, realistic, and timely (SMART) [16]. They have been shown to be successful in changing health behaviours in a multitude of settings and health disciplines [17, 18]. However, there are yet to be reported investigations of whether using synchronous compared to using asynchronous approaches for delivery of dietetic behaviour change interventions has a differential impact on goal achievement [19].

The present study is aimed at exploring whether the mode of nutrition intervention delivery (telephone or mobile app) affected participant goal achievement of an early and intensive nutrition intervention delivered to upper gastrointestinal cancer patients. It also examined the types of behaviour change techniques that were employed between study intervention groups and the relationships between participant demographics, dose of intervention, frequency of intervention delivery, and goal achievement. It was hypothesised that there would be no difference in exposure to behaviour change techniques and the achievement of nutrition care goals between the two intervention groups.

## 2. Methods

### 2.1. Study Design

This investigation took place within the context of a three-arm randomised controlled trial of two differing models of early, intensive nutrition intervention delivery: synchronous (telephone group) and asynchronous (mobile app group), compared to a standard care control group in patients with upper gastrointestinal (UGI) (oesophageal, gastric, and pancreatic) cancers.

The overall results from this trial have previously been reported and indicated that the two innovative models of nutrition care enabled much earlier commencement of nutrition intervention and contact with a dietitian, breaking down access barriers and providing a service in the comfort of participants' homes [8]. No differences were seen in quality of life measured using quality-adjusted life years lived between these two intervention groups and the control [8].

The Monash Health Human Research Ethics Committee (14th October 2016 HREC/16/MonH/290) granted ethical approval. Participants provided informed verbal consent to participate. The trial was registered prospectively on the Australian and New Zealand Clinical Trial Registry (Trial ID: ACTRN12617000152325 27th January 2017).

### 2.2. Setting

Participants were recruited from four health services (public and private) across southeast metropolitan Melbourne, Victoria, Australia. Participants who enrolled into the study received the intervention in addition to standard care, delivered to them at home by the research team.

### 2.3. Participants

Participants were those newly diagnosed (<4 weeks) with UGI cancer and planned to commence surgical and/or medical (chemotherapy and/or radiotherapy) cancer treatment.

### 2.4. Eligibility

Patients who had commenced chemotherapy or radiology were ineligible to participate in the trial. Those who had urgent surgical treatment, however, were deemed eligible. Recruitment occurred between April 2017 and July 2019. Screening for eligible patients occurred through weekly outpatient upper gastrointestinal clinic lists and multidisciplinary meetings by surgeons and ward dietitians. In person or telephone contact was made to eligible participants to invite participation in the study.

### 2.5. Randomisation and Blinding

An independent biostatistician was employed to complete a permuted block randomisation with two group stratification (Malnutrition Screening Tool (MST) score of <3 or ≥3) [20] performed using computer-generated random numbers (STATA version 14, StataCorp LP, College Station, TX, USA). Researchers who conducted recruitment, data collection, and analysis were blinded to participant group allocation. Opaque-sealed envelopes were used to conceal group allocation. The research dietitian (KF) revealed group allocation to the participant.

### 2.6. Interventions

Following participant's consent, recruitment, and baseline data collection, the research dietitian contacted participants with their randomisation information. This was done as soon as was practicable. The dietitian and participant then set a time and date for the completion of their initial nutrition assessment and commencement of the 18-week intervention period.

Participants received either a weekly or fortnightly intervention based on collaboration between the dietitian and patient, and their combined assessment of participant's current intensity requirements. If participants were uncontactable after two attempts or were hospitalised at the time of review, they did not receive their intervention for that week. There were no extensions to the 18-week period even if reviews were missed.

The Behaviour Change Technique Taxonomy v1 (BCTTv1) formed the basis for the behaviour change techniques used to support SMART goal setting (Table 1) [16]. A variety of behaviour change techniques, both routine and supplementary, were selected for use *ante hoc*. It has been shown that the more behaviour change techniques employed during the provision of eHealth interventions, the more effective they are in eliciting behaviour change [21].

### 2.7. Synchronous Telephone Intervention

Participants randomised to the telephone group received regular weekly or fortnightly phone contact from the research dietitian to their own home or mobile telephone.

### 2.8. Asynchronous Mobile Intervention

Participants were provided with a tablet computer (e.g., Apple iPad) and 6 months of wireless connectivity. A preexisting mobile app, myPace [22], was employed to deliver the asynchronous intervention. It allowed a messaging function for the participant and dietitian to communicate asynchronously, and daily reminders to assist with self-monitoring of weight and completion of scheduled small steps (goals) [23]. Participant nutritional intake, weight, and nutrition impact symptoms were used to prepare a tailored behavioural management program with participant engagement to empower and motivate change which was monitored and modified throughout the 18-week intervention period [21].

### 2.9. Participant Data

An independent, blinded researcher (LH) collected demographic (age, gender, cancer location, baseline malnutrition risk scores MST [20] and PG-SGA_SF_ [24]), anthropometrical (weight, height, BMI), health-related quality of life scores (EORTC-QLQ-C30 and EQ-ED-5L instruments) [25, 26], and survival data at baseline, 3, 6, and 12 months.

The research dietitian (KF) maintained a set of participant records, collating data from each week of intervention delivery. This data included (1) content, (2) dose/contact, (3) behaviour change, (4) barriers and facilitators to engagement, (5) acceptability, and (6) factors mediating engagement, behaviour, and health outcomes.

Topics for goal setting and the behaviour change techniques used to achieve these goals were also recorded from the dietetic consultations, either through interactions via telephone calls or via myPace asynchronous messaging and myPace Small Steps (goal setting). These topics for goal setting included nutrition optimisation, nutrition impact symptoms, and pharmacological and psychosocial support.

### 2.10. Data Analysis

Content analysis using a classification matrix was employed to explore the classification of goal setting and behaviour change techniques. Goal achievement was classified using a proportion-based approach rather than raw count data to ensure that it was classified using the same method between the two intervention arms. Goals were considered achieved when at least 50% of the goals set for the week were met. If the participant missed a fortnightly review, they received a zero for goal achievement. This data was analysed using negative binomial regression (based on variance) to determine the proportion of behaviour change goals achieved per participant compared between groups.

A Poisson regression model was used to predict the higher frequency of participation in the intervention. The relationships between demographics, dose (frequency), and goal achievement were explored through a multiple regression model. Demographic data included age, gender, cancer location, group, and baseline MST and PG-SGA SF scores. Univariate analysis exploring the relationship between demographic variables and goal achievement was completed. Relationships between demographics and goal achievement were also then explored using backward stepwise regression analysis as described by Hosmer and Lemeshow [27].

## 3. Results

A total of *n* = 111 participants were recruited from April 2017 to July 2019. There were *n* = 36 in the mobile app group and *n* = 38 in the telephone group (*n* = 37 in control group).

Participant baseline characteristics are presented in Table 2. There were no significant differences between the two intervention groups.

### 3.1. Withdrawal

A total of *n* = 10 participants withdrew either at baseline (after randomisation) or during the intervention period. There were three participants in the control group (8%), six in the electronic group (17%), and one in the telephone group (3%). The mobile app-based group had a higher risk of withdrawal from the intervention (RR 1.17, 95% CI 1.00 to 1.36) compared to the telephone group. Four individuals withdrew at randomisation due to group allocation, and two withdrew due to lack of engagement with the intervention in the mobile app group.

### 3.2. Contact with Dietitian

The participants in the telephone group had over 400 interventions delivered to them whilst the electronic group had 188, resulting in 1.99 times increased frequency of contact with the research dietitian (incidence rate ratio (IRR) 1.99, 95% CI 1.67 to 2.36, *p* = <0.001).

### 3.3. Goal Achievement

Participants in the telephone group had IRR 2.37 times greater proportion of goals achieved than the electronic group (95% CI 1.10 to 5.11, *p* = 0.03). Group allocation significantly impacted the type of behaviour change techniques that were employed with participants. The mobile app group were less likely to be exposed to eight of the 24 different techniques whilst having higher exposure to information on antecedents, information about health consequences, and social support (practical) (Table 3).

### 3.4. Relationship Analyses

Analysis exploring relationships between participant demographics, dose, timing, and frequency and whether the goal set was achieved found that those in the mobile app group achieved fewer goals compared to the telephone group (-0.14, 95% CI: -0.19 to -0.07, *p* = <0.001). The dose of intervention (i.e., the number of reviews with the dietitian) was positively associated with participant goal achievement in the telephone group (0.03, 95% CI 0.14 to 0.08, *p* = <0.001).

Univariate analysis exploring relationships between participant demographics (age, gender, cancer type, weight, MST score, PG-SGA_SF_ score, EORTC-QLQ-C30 Utility Score, EQ-5D-5L Utility Score), group allocation, and goal achievement revealed that telephone group allocation was positively associated with goal achievement (Table 4).

Multiple regression analysis explored relationships between participant demographics (age, gender, cancer type, weight, MST score, PG-SGA_SF_ score, EORTC-QLQ-C30 Utility Score, EQ-5D-5L Utility Score), group allocation, and goal achievement and revealed that telephone group allocation and male gender were positively associated with goal achievement (Table 5).

## 4. Discussion and Conclusion

### 4.1. Discussion

In a cohort of newly diagnosed individuals with upper GI cancers, synchronous delivery of early and intensive nutrition care via the telephone led to more contact with the research dietitian, greater numbers of behaviour change techniques being employed, and greater proportion of goals achieved compared to the asynchronous, mobile app group. The telephone group were more likely to be exposed to eight different behaviour change techniques than the mobile app group. The mobile app group had significantly lower doses of intervention delivered, and behaviour change techniques were employed which led to less goal setting and achievement. Information sharing in this group was also very different from the telephone group, as there was a reliance on participants to disclose information without the dietitian to prompt them immediately, for example, when discussing treatment side effects. Participants in the mobile app group often did not answer questions that were embedded into the original message from the dietitian. The observation that mode of delivery can impact on the breadth and depth of information sharing has previously been reported for delivery of electronic medical consultations (e-visits) [28]. Insufficient information provision has also been reported to impact formation of diagnoses stemming from asynchronous communication modalities [29].

The dose received and fewer goals achieved in the asynchronous, mobile app group in the present study suggest that mobile app technology does not support engagement in a way that is equivalent to synchronous, telephone-based interactions. A recent scoping review of mobile applications for managing symptoms of palliative cancer patients at home found that they improved the provision of care predominantly through education and knowledge attainment of symptom management, with a focus on family inclusion [30]. This study found that a variety of application systems need to be used to improve accessibility and engagement such as videos, avatars, cultural integration, and emergency call features, which were lacking in the mobile app used in this intervention. In a recent systematic review and thematic analysis exploring which design features improve engagement with mobile health interventions [31], a checklist of 29 items reported to enhance user engagement was developed which included aesthetic, navigation, personalisation, reinforcement, communication, message presentation, and credibility.

The design of interventions needs to encompass the breadth of techniques required for improved engagement including aesthetics and functionality such as larger font size, videos, interactive modules, and direct healthcare communication, whilst being personalised and tailored for each individual. The myPace mobile app was originally codesigned with consumers and dietitians for weight management [22]. It connected dietitians and patients through personalised monitoring features called “small steps” acting as goals to achieve which dietitians could respond to regularly to promote ongoing motivation [23]. A small pilot study (unpublished) examining the use of myPace in upper gastrointestinal cancer patients suggested that the mobile app might be useful given the underpinning of behaviour change theory and techniques to support goal achievement.

We acknowledge a number of limitations with this research study. Data collection utilised subjective patient-reported outcomes which may be affected by the participants' perceptions and attitudes, as well as external factors such as socioeconomic status, which may influence outcomes [32]. Some of the data presented is liable to bias as participants may have experienced a positive relationship with the dietitian and therefore were more likely to report obsequious responses. We also recognise that many patients in this study had a life-limiting disease and may have responded differently to other patient groups where survival is high. The high numbers of withdrawals from the mobile app group are also a limitation but provide important information on patient preferences and insights into how they would like to receive their nutrition care. Participants were not blinded and may have inadvertently exposed their group allocation to the blinded data collector during 3-, 6-, and 12-month data collection despite an independent blinded researcher collecting baseline and follow-up data. The research dietitian was also not blinded, and this may have biased the way in which the interventions were delivered.

### 4.2. Practice Implications

The study has highlighted several future research directions that may be of value for optimising service delivery using synchronous and/or asynchronous approaches. Examining different populations, their characteristics, and motivators to use eHealth technologies should be explored more widely. Codesign may be a potential strategy to enable this.

### 4.3. Conclusion

Telephone nutrition intervention delivery led to a higher frequency of goal achievement compared to the mobile app intervention. There was also a higher number of behaviour change techniques employed which may have facilitated the greater goal achievement. Mobile app-based delivery may have poorer acceptance in this population with high levels of withdrawal. We need to ensure that specifically designed technologies for our target populations continue to be embedded into the care of our patients into the future whilst ensuring they are fit for purpose, efficacious, acceptable to both patients and healthcare providers, and cost-effective.

I confirm that all patient/personal identifiers have been removed or disguised, so the patient/persons described are not identifiable and cannot be identified through the details of the story.

## Figures and Tables

**Table 1:** Behaviour change techniques [16].

Behaviour change technique	Definition [16]	Example	Classification^a^
*1. Goals and planning*			
1.1 Goal setting (behaviour)	Set or agree on a goal defined in terms of the behaviour to be achieved	Set the goal of eating 5 pieces of fruit per day	Routinely used
1.2 Problem-solving	Analyse or prompt the person to analyse factors influencing the behaviour and generate or select strategies that include overcoming barriers and/or increasing facilitators	Prompt the patient to identify potential barriers to them drinking a particular supplement (e.g., bad taste) and discuss ways in which they could overcome them (e.g., mix with strawberries)	Supplementary
1.3 Goal setting (outcome)	Set or agree on a goal defined in terms of a positive outcome of wanted behaviour	Set a weight gain goal (e.g., 0.5 kilogram over one week) as an outcome of changed eating patterns	Supplementary
1.4 Action planning	Prompt detailed planning of performance of the behaviour (must include at least one of context, frequency, duration, and intensity). Context may be environmental (physical or social) or internal (physical, emotional, or cognitive)	Prompt planning the drinking of a supplement at a particular time (e.g., before work) on certain days of the week	Routinely used
1.5 Review goal (behaviour)	Review behaviour goal(s) jointly with the person and consider modifying goal(s) or behaviour change strategy in light of achievement. This may lead to resetting the same goal, a small change in that goal or setting a new goal instead of (or in addition to) the first, or no change	Ask if the patient drank the supplement as planned	Routinely used
1.6 Highlight discrepancy between current and goal (behaviour or outcome)	Draw attention to discrepancies between a person's current behaviour (in terms of the form, frequency, duration, or intensity of that behaviour) or outcome and the person's previously set behavioural goals or action plans	Point out that the recorded supplement intake fell short of the goal set	Routinely used
1.7 Review goal (outcome)	Review outcome goal(s) jointly with the person and consider modifying goal(s) in light of achievement. This may lead to resetting the same goal, a small change in that goal or setting a new goal instead of, or in addition to the first	Ask if the patient achieved the weight gain goal	Supplementary
*2. Feedback and monitoring*			
2.1 Monitoring of behaviours by others, without feedback	Observe or record behaviour with the person's knowledge as part of a behaviour change strategy	Have the partner observe food intake behaviours and make notes on content and frequency	Supplementary
2.3 Self-monitoring of behaviour	Establish a method for the person to monitor and record their behaviour(s) as part of a behaviour change strategy	Ask the person to record daily, in a diary, the amount of food that they have eaten	Supplementary
2.4 Self-monitoring of outcome of behaviour	Establish a method for the person to monitor and record the outcome(s) of their behaviour as part of a behaviour change strategy	Ask the person to weigh themselves at the end of each day, over a two-week period, and record their daily weight on a graph to increase food intake	Supplementary
2.5 Monitoring outcomes of behaviours by others, without feedback	Observe or record outcomes of behaviour with the person's knowledge as part of the behaviour change strategy	Record weight maintenance/gain, blood glucose levels	Supplementary
2.6 Biofeedback	Provide feedback about the body using an external monitoring device as part of a behaviour change strategy	Inform the person of the blood sugar levels to improve their adoption of insulin use	Supplementary
2.7 Feedback on outcome(s) of behaviour	Monitor and provide feedback on the outcome of the performance of the behaviour	Inform the person of their stable weight following implementation of high energy, high protein diet regimen	Supplementary
*3. Social support*			
3.1 Social support (unspecified)	Advise on, arrange, or provide social support (e.g., from friends, relatives, colleagues, “buddies,” or staff) or noncontingent praise or reward for performance of the behaviour. It includes encouragement and counselling, but only when it is directed at the behaviour	Arrange for a partner to encourage patient to use supplements	Supplementary
3.2 Social support (practical)	Advise on, arrange, or provide practical help (e.g., from friends, relatives, colleagues, “buddies,” or staff) for performance of the behaviour	Ask the partner to mix the supplement with strawberries for the patient	Supplementary
3.3 Social support (emotional)	Advise on, arrange, or provide emotional social support (e.g., from friends, relatives, colleagues, buddies, or staff) for performance of behaviour	Ask a patient to take a partner to their surgeon's appointment	Supplementary
*4. Shaping knowledge*			
4.1 Instruction on how to perform behaviour	Advise or agree on how to perform the behaviour (includes “Skills training”)	Demonstrate or describe to the person how to prepare thickened fluids	Routinely used
4.2 Information about antecedents	Provide information about antecedents (e.g., social and environmental situations and events, emotions, cognitions) that reliably predict performance of the behaviour	Discuss how people find it difficult to follow their diet when they attend social events	Supplementary
*5. Natural consequences*			
5.1 Provide information (e.g., written, verbal, visual) about health consequences of performing the behaviour	Provide information (e.g., written, verbal, visual) about health consequences of performing the behaviour	Present written information about the positive effect on weight and maintaining nutrition status with adoption of a high energy high protein diet regimen	Supplementary
*6. Associations*			
6.1 Prompts/cues	Introduce or define environmental or social stimulus with the purpose of prompting or cueing the behaviour. The prompt or cue would normally occur at the time or place of performance	Put a sticker on the fridge to avoid eating cheesecake	Supplementary
*7. Repetition and substitution*			
7.1 Graded tasks	Set easy-to-perform tasks, making them increasingly difficult, but achievable, until behaviour is performed	Ask patient to consume supplement once per day the first week, then twice per day the second week.	Supplementary
*8. Comparison of outcomes*			
8. 1 Consider pros and cons	Advise the person to identify and compare reasons for wanting (pros) and not wanting (cons) to change the behaviour	Advise the person to list and compare the advantages and disadvantages of drinking the supplement	Supplementary
*9. Regulation*			
9.1 Pharmacological support	Provide or encourage the use of or adherence to drugs to facilitate behaviour change	Advise the person to take regular antinausea medications when they are nauseated	Supplementary
*10. Antecedents*			
10.1 Restructuring the physical environment	Change or advise to change the physical environment in order to facilitate performance of the wanted behaviour	Advise to make a 1 L jug of Sustagen and keep in the fridge to sip during the day	Supplementary
10.2 Restructuring the social environment	Change or advise to change the social environment in order to facilitate performance of the wanted behaviour	Advise the person to sit with a family member/friend at meals and snacks	Supplementary
10.3 Body changes	Alter body structure, functioning, or support directly to facilitate behaviour change	Prompt use of dentures to promote food consumption	Supplementary
*11. Self-belief*			
11.1 Verbal persuasion about capability	Tell the person that they can successfully perform the wanted behaviour, arguing against self-doubts, and asserting that they can and will succeed	Tell the person that they can successfully maintain their weight despite ongoing treatment	Supplementary
11.2 Focus on past success	Advise to think about or list previous successes in performing the behaviour	Advise to describe or list the times that they were able to drink their prescribed nutrition supplement drinks during chemotherapy	Supplementary

^a^Behaviour change techniques have been classified as routinely used techniques to be used with all participants, and supplementary techniques that can optionally be used.

**Table 2:** Demographic data at baseline by intervention group.

Demographic data	Mobile app (*n* = 36)	Telephone (*n* = 38)
Age year mean (sd)	66.6 (9.7)	67.5 (10.3)
Gender *n* (%)		
Male	26 (72%)	25 (66%)
Female	10 (28%)	13 (34%)
Cancer type *n* (%)		
Oesophageal	17 (47%)	16 (42%)
Pancreatic	10 (28%)	18 (47%)
Gastric	9 (25%)	4 (11%)
Weight mean (sd)	76.4 (14.7)	71.9 (12.7)
MST score mean (sd)	2.78 (1.94)	2.72 (2.18)
PG-SGA_SF_ score mean (sd)	8.5 (6.5)	8.5 (6.2)
EQ-5D-5L–median (IQR)		
Mobility (0-100, higher scores equal more difficulty with task completion)	1 (1.3)	1 (1.1)
Self-care (0-100, higher scores equal more difficulty with task completion)	1 (1.1)	2 (1.2)
Usual activities (0-100, higher scores equal more difficulty with task completion)	2 (1.2)	1 (1.1)
Pain or discomfort (0-100, higher scores equal higher severity of symptoms)	1 (1.1)	1 (1.2)
Anxiety or depression (0-100, higher scores equal higher severity of symptoms)	2 (1.2)	2 (1.3)
EQ-5D-5L-utility score–mean (sd) (0-100, higher scores better QoL)	0.65 (0.20)	0.71 (0.23)
EQ-5D-5L-visual analogue scale–mean (sd) (0-100, higher scores better QoL)	62.08 (22.01)	65.04 (22.9)
EORTC QLQ-C30 score–mean (sd)		
Global health (0-100, higher scores better QoL)	63.51 (36.61)	79.28 (25.72)
Physical functioning (0-100, higher scores better QoL)	72.52 (29.71)	83.33 (21.52)
Role functioning (0-100, higher scores better QoL)	27.93 (27.51)	10.81 (21.59)
Emotional functioning (0-100, higher scores better QoL)	26.13 (30.60)	46.85 (36.39)
Cognitive functioning (0-100, higher scores better QoL)	81.23 (20.08)	63.41 (26.17)
Social functioning (0-100, higher scores better QoL)	85.09 (18.90)	72.15 (21.26)
Fatigue (0-100, higher scores more symptoms)	11.84 (20.47)	35.38 (29.21)
Nausea and vomiting (0-100, higher scores more symptoms)	28.95 (29.17)	13.16 (23.94)
Pain (0-100, higher scores more symptoms)	61.22 (24.60)	17.54 (28.72)
Dyspnoea (0-100, higher scores more symptoms)	73.38 (25.34)	65.28 (35.04)
Insomnia (0-100, higher scores more symptoms)	42.90 (31.44)	74.07 (32.96)
Appetite loss (0-100, higher scores more symptoms)	12.96 (18.30)	26.63 (33.36)
Constipation (0-100, higher scores more symptoms)	22.22 (31.87)	28.70 (35.77)
Diarrhoea (0-100, higher scores more symptoms)	7.02 (22.13)	10.81 (22.30)
Financial difficulties (0-100, higher scores more symptoms)	16.66 (40.19)	4.63 (19.76)

EQ-5D-5L: EuroQol 5D-5L instrument [26]; EORTC QLQ-C30: European Organization for Research and Treatment of Cancer Quality of Life Question–Core 30 [25]; PG-SGA: Patient-Generated Subjective Global Assessment [24]; sd: standard deviation; MST: Malnutrition Screening Tool [20]; LGTBQI+: lesbian, gay, transexual, bisexual, queer, intersex+.

**Table 3:** The frequency of behaviour change techniques used by the research dietitian to support goal achievement between intervention groups.

Behaviour change techniques	Mobile app (*n* = 36)	Telephone (*n* = 38)	RR (95% CI)
1.1. Goal setting	33 (92%)	38 (100%)	0.92 (0.83-1.01)
1.2 Problem-solving	33 (92%)	38 (100%)	0.92 (0.83-1.01)
1.3 Goal setting (outcome)	33 (92%)	38 (100%)	0.92 (0.83-1.01)
1.4 Action planning	33 (92%)	38 (100%)	0.92 (0.83-1.01)
1.5 Review behaviour (goal)	24 (67%)	38 (100%)	0.66 (0.53 to 0.84)
1.6 Discrepancy between current behaviour and goal	11 (31%)	0 (0%)	.
1.7 Review outcome (goal)	23 (64%)	37 (97%)	0.66 (0.51 to 0.84)
2.2 Feedback on behaviour	25 (69%)	37 (97%)	0.71 (0.57 to 0.89)
2.3 Self-monitoring of behaviour	33 (92%)	37 (97%)	0.94 (0.84 to 1.05)
2.4 Self-monitoring of outcome of behaviour	33 (92%)	37 (97%)	0.94 (0.84 to 1.05)
2.6 Biofeedback	1 (3%)	0 (0%)	.
2.7 Feedback on outcomes of behaviour	25 (69%)	37 (97%)	0.71 (0.57 to 0.89)
3.2 Social support (practical)	11 (31%)	5 (13%)	2.32 (0.89 to 6.02)
3.3 Social support (emotional)	2 (6%)	7 (18%)	0.30 (0.07 to 1.36)
4.1 Instruction on how to perform behaviour	33 (92%)	38 (100%)	0.92 (0.83-1.01)
4.2 Information on antecedents	17 (47%)	3 (8%)	5.98 (1.91 to 18.69)
5.1 Information about health consequences	33 (92%)	33 (87%)	1.06 (0.90 to 1.24)
7.1 Prompts/cues	33 (92%)	0 (0%)	.
8.7 Graded tasks	22 (61%)	32 (84%)	0.73 (0.54 to 0.97)
9.1 Credible source	33 (92%)	38 (100%)	0.92 (0.83-1.01)
9.2 Pros and cons	8 (22%)	0 (0%)	.
11.1 Pharmacological support	14 (42%)	27 (71%)	0.55 (0.35 to 0.86)
15.1 Verbal persuasion of capability	8^∗^ (22%)	33 (87%)	0.26 (0.14 to 0.48)
15.3 Focus on past success	9 (25%)	31 (82%)	0.31 (0.17 to 0.55)

^∗^Initial assessment completed by phone for both groups.

**Table 4:** Univariate analyses of demographic variables predicting goal achievement.

Goal achievement	Coefficient	Std err	*t*	95% CI	95% CI	*p*
Group	0.41	0.06	7.37	0.31	0.53	<0.001
Age	0.01	0.01	0.17	-0.01	0.01	0.86
Gender	0.15	0.08	1.32	-0.05	0.26	0.19
Cancer type	-0.06	0.05	-1.10	-0.16	0.05	0.28
Weight	-0.02	0.03	-0.70	-0.01	0.01	0.49
MST score	-0.12	0.18	-0.66	-0.05	0.02	0.51
PG-SGA_SF_ score	-0.01	0.01	-0.52	-0.15	0.01	0.60
EORTC-QLQ-C30 Utility Score	0.19	0.17	1.14	-0.15	0.53	0.26
EQ-5D-5L Utility Score	0.15	0.15	1.02	-0.15	0.46	0.31

EQ-5D-5L: EuroQol 5D-5L instrument [26]; EORTC QLQ-C30: European Organization for Research and Treatment of Cancer Quality of Life Question–Core 30 [25]; PG-SGA: Patient-Generated Subjective Global Assessment [24]; sd: standard deviation; MST: Malnutrition Screening Tool [20].

**Table 5:** Multiple regression analyses of demographic data predicting goal achievement.

Goal achievement	Coefficient	Std err	*t*	95% CI	95% CI	*p*
Group	0.42	0.05	7.73	0.31	0.53	<0.001
Gender	0.14	0.06	2.31	0.02	0.25	0.02
_cons	0.12	0.06	2.13	0.01	0.24	0.04

_cons: constant.

## Data Availability

Nonidentifiable aggregate data may be requested by contacting the corresponding author.

## Abbreviations

EORTC QLQ-C30: European Organization for Research and Treatment of Cancer Quality of Life Questionnaire-Core 30
ED 5Q 5L: EuroQol Group 5D-5L instrument
MST: Malnutrition Screening Tool (malnutrition risk assessment tool)
QoL: Quality of life
PG-SGA_SF_: Patient-Generated Subjective Global Assessment Short Form (malnutrition risk assessment tool)
UGI: Upper gastrointestinal
BMI: Body mass index.

## References

[1] Andreyev H. J. N., Norman A. R., Oates J., Cunningham D. (1998). Why do patients with weight loss have a worse outcome when undergoing chemotherapy for gastrointestinal malignancies?. *European Journal of Cancer*.

[2] Gavazzi C., Colatruglio S., Sironi A., Mazzaferro V., Miceli R. (2011). Importance of early nutritional screening in patients with gastric cancer. *The British Journal of Nutrition*.

[3] Reim D., Friess H. (2016). Feeding challenges in patients with esophageal and gastroesophageal cancers. *Gastrointestinal Tumors*.

[4] Choi W. J., Kim J. (2016). Nutritional care of gastric cancer patients with clinical outcomes and complications: a review. *Clinical Nutrition Research*.

[5] Sandrucci S., Beets G., Braga M., Dejong K., Demartines N. (2018). Perioperative nutrition and enhanced recovery after surgery in gastrointestinal cancer patients. A position paper by the ESSO task force in collaboration with the ERAS society (ERAS coalition). *European Journal of Surgical Oncology*.

[6] Loeliger J., Kiss N. (2015). *Phase II Malnutrition in Victorian Cancer Services: Summary Report*.

[7] Hanna L., Huggins C. E., Furness K. (2018). Effect of early and intensive nutrition care, delivered via telephone or mobile application, on quality of life in people with upper gastrointestinal cancer: study protocol of a randomised controlled trial. *BMC Cancer*.

[8] Huggins C. E., Hanna L., Furness K. (2022). Effect of early and intensive telephone or electronic nutrition counselling delivered to people with upper gastrointestinal cancer on quality of life: a three-arm randomised controlled trial. *Nutrients*.

[9] Wosik J., Fudim M., Cameron B. (2020). Telehealth transformation: COVID-19 and the rise of virtual care. *Journal of the American Medical Informatics Association*.

[10] World Health Organization (WHO) (2011). *mHealth: New Horizons for Health through Mobile Technology*.

[11] Verhoeven F., Tanja-Dijkstra K., Nijland N., Eysenbach G., van Gemert-Pijnen L. (2010). Asynchronous and synchronous teleconsultation for diabetes care: a systematic literature review. *Journal of Diabetes Science and Technology*.

[12] Wootton R. (2012). Twenty years of telemedicine in chronic disease management – an evidence synthesis. *Journal of Telemedicine and Telecare*.

[13] Kern J. (2006). Evaluation of teleconsultation systems. *International Journal of Medical Informatics*.

[14] Michie S., van Stralen M. M., West R. (2011). The behaviour change wheel: a new method for characterising and designing behaviour change interventions. *Implementation Science*.

[15] Moore G. F., Audrey S., Barker M. (2015). Process evaluation of complex interventions: Medical Research Council guidance. *BMJ*.

[16] Michie S., Richardson M., Johnston M. (2013). The behavior change technique taxonomy (v1) of 93 hierarchically clustered techniques: building an international consensus for the reporting of behavior change interventions. *Annals of Behavioral Medicine*.

[17] Davidson K. W., Goldstein M., Kaplan R. M. (2003). Evidence-based behavioral medicine: what is it and how do we achieve it?. *Annals of Behavioral Medicine*.

[18] Bonell C., Fletcher A., Morton M., Lorenc T., Moore L. (1982). Realist randomised controlled trials: a new approach to evaluating complex public health interventions. *Social Science & Medicine*.

[19] Furness K., Sarkies M. N., Huggins C. E., Croagh D., Haines T. P. (2020). Impact of the method of delivering electronic health behavior change interventions in survivors of cancer on engagement, health behaviors, and health outcomes: systematic review and meta-analysis. *Journal of Medical Internet Research*.

[20] Ferguson M., Capra S., Bauer J., Banks M. (1999). Development of a valid and reliable malnutrition screening tool for adult acute hospital patients. *Nutrition*.

[21] Webb T. L., Joseph J., Yardley L., Michie S. (2010). Using the internet to promote health behavior change: a systematic review and meta-analysis of the impact of theoretical basis, use of behavior change techniques, and mode of delivery on efficacy. *Journal of Medical Internet Research*.

[22] Harricharan M., Gemen R., Celemín L. F. (2015). Integrating mobile technology with routine dietetic practice: the case of myPace for weight management. *Proceedings of the Nutrition Society*.

[23] Barnett J., Harricharan M., Fletcher D., Gilchrist B., Coughlan J. (2015). myPace: an integrative health platform for supporting weight loss and maintenance behaviors. *IEEE Journal of Biomedical and Health Informatics*.

[24] Bauer J., Capra S., Ferguson M. (2002). Use of the scored patient-generated subjective global assessment (PG-SGA) as a nutrition assessment tool in patients with cancer. *European Journal of Clinical Nutrition*.

[25] Aaronson N. K., Ahmedzai S., Bergman B. (1993). The European Organization for Research and Treatment of Cancer QLQ-C30: a quality-of-life instrument for use in international clinical trials in oncology. *JNCI Journal of the National Cancer Institute*.

[26] Herdman M., Gudex C., Lloyd A. (2011). Development and preliminary testing of the new five-level version of EQ-5D (EQ-5D-5L). *Quality of Life Research*.

[27] Hosmer D. W., Lemeshow S. (2000). *Applied Logistic Regression*.

[28] Nguyen O. T., Alishahi Tabriz A., Huo J., Hanna K., Shea C. M., Turner K. (2021). Impact of asynchronous electronic communication-based visits on clinical outcomes and health care delivery: systematic review. *Journal of Medical Internet Research*.

[29] Yau G. L., Williams A. S., Brown J. B. (2011). Family physicians' perspectives on personal health records: qualitative study. *Canadian Family Physician*.

[30] Putranto D., Rochmawati E. (2020). Mobile applications for managing symptoms of patients with cancer at home: a scoping review. *International Journal of Nursing Practice*.

[31] Wei Y., Zheng P., Deng H., Wang X., Li X., Fu H. (2020). Design features for improving mobile health intervention user engagement: systematic review and thematic analysis. *Journal of Medical Internet Research*.

[32] Kluzek S., Dean B., Wartolowska K. A. (2022). Patient-reported outcome measures (PROMs) as proof of treatment efficacy. *BMJ Evidence-Based Medicine*.

[33] Furness K., Huggins C. E., Hanna L., Croagh D., Sarkies M., Haines T. P. (2023). *Comparison of goal achievement during an early, intensive nutrition intervention delivered to people with upper gastrointestinal cancer by telephone compared with mobile application*.

